# Emerging Trends in Integrated Digital Microfluidic Platforms for Next-Generation Immunoassays

**DOI:** 10.3390/mi15111358

**Published:** 2024-11-08

**Authors:** Kaixin Su, Jiaqi Li, Hailan Liu, Yuan Zou

**Affiliations:** 1 Key Laboratory of Clinical Laboratory Diagnostics (Ministry of Education), College of Laboratory Medicine, Chongqing Medical University, Chongqing 400016, China; sukaixin@stu.cqmu.edu.cn (K.S.); lijiaqi@stu.cqmu.edu.cn (J.L.); liuhailan@stu.cqmu.edu.cn (H.L.); 2 Western (Chongqing) Collaborative Innovation Center for Intelligent Diagnostics and Digital Medicine, Chongqing 401329, China

**Keywords:** digital microfluidics (DMF), immunoassay, integration, point-of-care testing (POCT)

## Abstract

Technologies based on digital microfluidics (DMF) have made significant advancements in the automated manipulation of microscale liquids and complex multistep processes. Due to their numerous benefits, such as automation, speed, cost-effectiveness, and minimal sample volume requirements, these systems are particularly well suited for immunoassays. In this review, an overview is provided of diverse DMF manipulation platforms and their applications in immunological analysis. Initially, droplet-driven DMF platforms based on electrowetting on dielectric (EWOD), magnetic manipulation, surface acoustic wave (SAW), and other related technologies are briefly introduced. The preparation of DMF is then described, including material selection, fabrication techniques and droplet generation. Subsequently, a comprehensive account of advancements in the integration of DMF with various immunoassay techniques is offered, encompassing colorimetric, direct chemiluminescence, enzymatic chemiluminescence, electrosensory, and other immunoassays. Ultimately, the potential challenges and future perspectives in this burgeoning field are delved into.

## 1. Introduction

Immunoassay is recognized as a sensitive and specific analytical tool used for quantifying analytes, predominantly proteins, in solution by leveraging antigens, antibodies, immune cells, and their secreted cytokines [1]. Common immunoassay techniques include enzyme-linked immunosorbent assay (ELISA) [2], radioimmunoassay (RIA) [3], fluorescence immunoassay (FIA) [4], and chemiluminescence immunoassay (CLIA) [5]. According to an in vitro diagnostics (IVD) analysis report published in 2023, immunoassays possess the most significant market size and constitute a pivotal segment of in vitro diagnostics [6].

In various fields such as clinical research, life sciences, and medicine, immunoassays have evolved as a prevalent testing modality, which is divided into two primary categories [7] based on the analytical process: homogeneous and heterogeneous assays. Heterogeneous assays, particularly sandwich assays, have become commonly used quantitative methods [8,9]. Nevertheless, traditional immunoassays present several challenges. Firstly, the entire process is cumbersome and comprises complex steps. Secondly, each binding step is limited by the diffusion time of the molecules to the surface, resulting in a lengthy assay duration. Thirdly, the assay necessitates substantial quantities of costly reagents, leading to elevated expenses. Lastly, the experimental apparatus is unwieldy and large, impeding the application of immunoassays in POCT.

In 1990, Manz et al. [10] proposed Micro Total Analysis Systems (*µ*TASs). Since then, microfluidics has been widely recognized as a rapidly emerging and innovative research field, becoming an integral part of many bioengineering and medical applications [11,12,13]. Generally, microfluidics can accurately control and manipulate the behavior of small quantities of fluid in integrated microfluidic devices. Based on the method of fluid manipulation, microfluidics can be subdivided into two main categories: continuous-flow microfluidics [14] and digital microfluidics (DMF) [15]. Continuous-flow microfluidics rely on a device with a special mechanical component, which is designed to meet the specific requirements of a particular application, often necessitating external equipment. Conversely, DMF is part of the droplet-based microfluidics technology; it does not depend on continuous liquid flow and eliminates micro-pumps, micro-valves, and complex three-dimensional (3D) flow channels. The digital microfluidic system consists of three key components [16,17]: the DMF chip, the drive and control unit, and the fluid and reagent management system. The DMF chip guides, mixes, separates, stores, and manipulates fluids to perform complex fluid dynamics processes. The drive and control unit provides the necessary power to move fluids and precisely regulates fluid flow. Currently, EWOD [18] is considered to be the most commonly used actuation strategy, followed by magnetic [19] and SAW actuation [20]. Various strategies have their own advantages and disadvantages. EWOD can easily separate and dispense droplets and provide automated and precise droplet control. As for magnetic actuation, what makes it unique is the dual function of magnetic particles. In addition to being able to drive droplets, the magnetic particles provide a functional solid substrate for molecular adsorption. For SAW control, the process of preparing sound wave equipment is relatively simple, so it can be easily integrated. As a technique for manipulating individual droplets, DMF offers simplified equipment, enhanced practicality, and improved stability [21].

DMF components have the potential to integrate multiple functions in a single device, and their integration into a single chip has promoted the development of lab-on-a-chip (LOC) [22], *µ*TAS [23], and POCT devices [24]. POCT is a technology that performs medical testing at the patient’s side. As a promising in vitro diagnostic method, POCT simplifies the traditional laboratory testing process, bringing it directly to the point of care. This allows clinicians to obtain results quickly, facilitating timely and informed decision-making [21,25]. POCT devices are complex systems because they integrate all the testing steps from “sample to answer”. The POCT system consists of a user interface, a sample delivery device, a reagent storage tank, a reaction tank, a detection system, a control system, and a data management system [26,27]. The advantages of DMF as a promising candidate for POCT applications can be summarized in the following three aspects. Firstly, DMF reduces the consumption of reagents and samples. One significant challenge in bioanalysis is the high cost of reagents and limited sample availability. DMF can produce droplets at picolitre to microliter levels, thus increasing the efficiency of the samples and reagents usage. Secondly, DMF reduces detection and analysis time. It is not only because of the lack of diffusion dynamics in the reaction with small liquid volumes but also because of faster and more efficient mixing of droplets by dynamic DMF operations. Thirdly, DMF enables automation through the use of programmed operations. The DMF equipment has a signal control system to automatically move the drops, so it can be used for multiple steps that need to be repeated. This automation capability further enhances the practicality and efficiency of DMF in POCT applications.

In recent years, tens of thousands of researchers have innovatively combined DMF and immunoassays to dramatically improve the shortcomings of traditional immunoassays [28,29]. Various research teams have approached this challenge from different angles: simplifying assay steps [28], integrating systems [30,31], and enhancing sensitivity [32,33]. For example, David R. Walt’s group [34] at Harvard University integrated DMF and droplet ELISA to improve the efficiency of sample use. Hsu, Zhang et al. [35,36,37] improved the sensitivity of interleukin-6 (IL-6), interleukin-8 (IL-8), and Granulocyte-macrophage colony-stimulating factor (GM-CSF) detection by adjusting the spatial structure, physical properties, and number of solid-phase carrier magnetic beads used. Yue et al. [38] introduced a breakthrough system called Poison Distribution Magnetic Bead Ordered Arrangement of Droplet (BOAD), in which each microdroplet is a separate reaction chamber with great potential for high-throughput detection. Meanwhile, various researchers have made concerted efforts to improve the limit of detection (LOD) for diverse protein biomarkers in clinical settings, yielding promising outcomes. Through these innovative approaches, the detection of biomarkers has been greatly enhanced. Some examples of tumors include the prostate-specific antigen (PSA), which indicates the health of the prostate, the alpha-fetoprotein (AFP), which indicates liver function, and the exosomes released by tumor cells, which disclose malignant activity. As with the immune response, the test has refined the identification of cytokines, allowing for the precise monitoring of γ-interferon (INF-γ) and interleukin-2 (IL-2), which regulate cell development, and tumor necrosis factor-α (TNF-α) and interleukin-1β (IL-1β), which are involved in inflammatory processes. Moreover, there have been notable advances in the identification of pathogenic antigens, as evidenced by the improved nucleoprotein detection of influenza A [34,38,39,40,41,42,43]. Consequently, portable and all-inclusive immunoassay analyzers that offer optimal integration and superior sensitivity have gradually emerged. Examples include the M2a/M5a microfluidic magnetic bead-based chemiluminescence immunoassay analyzer from Huamaxing Micro, the mLabs microfluidic fluorescence immunoassay analyzer from Micropoint Bio, and the Ella fully automated microfluidic immunoassay analyzer from ProteinSimple, a division of Bio-Techne Group, USA. These advancements represent a major step forward in immunoassay techniques, paving the way for more precise and efficient diagnostic tools.

This manuscript provides an overview of the various manipulation platforms for DMF and their applications in immunological analysis. Initially, we present a succinct introduction to EWOD-based droplet-driven DMF platforms, magnetic-based droplet-driven DMF platforms, SAW-based droplet-driven DMF platforms, and other DMF platforms. The preparation of DMF is then described, including material selection, fabrication techniques and droplet generation. After that, we detail advancements in integrating digital microfluidics with various assay technologies in immunoassays, encompassing colorimetric, direct chemiluminescence, enzymatic chemiluminescence, electrosensory assays, as well as other immunoassays. Last but not least, we discuss the potential challenges and future perspectives in the application of these technologies in the field.

## 2. Role of Digital Microfluidics

Digital microfluidic platforms enable sample preparation, mixing, separation, and detection within a single chip, offering a versatile solution for immunoassays. These platforms have found widespread applications in the detection of antigens and antibodies for infectious diseases [44,45], hormone assays [46,47], tumor-related molecular diagnostics [48], and various other detections [49,50]. The accurate manipulation of samples is one of the key factors for the efficient operation of the platform. Currently, depending on the operating principles, digital microfluidic platforms can be categorized as EWOD droplet-driven DMF, magnetic droplet-driven DMF, surface acoustic wave (SAW) droplet-driven DMF, and other droplet-driven DMFs. Each platform exhibits distinct advantages and disadvantages. In this section, we aim to provide a comprehensive description of these various DMF platforms, emphasizing their respective capabilities and limitations.

### 2.1. Electrowetting on Dielectric (EWOD) Droplet-Driven DMF

EWOD has emerged as a robust biological and chemical diagnostic technique, offering advantages such as small sample size, high sensitivity, high throughput, and automation capabilities. The implementation of EWOD technology not only minimizes costs but also reduces the system’s footprint, thus enabling miniaturization and portability of the detection system [51,52]. In 1875, Lippmannet et al. [53] made the seminal discovery of the electrowetting phenomenon, which describes the spreading of conductive liquids on electrode surfaces under the influence of an applied electric field. Subsequently, in 2000, DMF based on EWOD was introduced by Pollack [54] and Lee [55]. This technique makes use of electrowetting on an electrically insulating substrate to avoid electrolysis, which leads to a more pronounced electrowetting effect before breakdown. Nowadays, it finds widespread applications across various fields, highlighting its versatility and reliability in modern scientific endeavors [56].

Based on plate configuration, DMF devices can be classified into single-plate [57] and two-plate formats [58]. Single-plate devices feature an open structure with only a lower substrate, whereas two-plate devices comprise two parallel plates, a lower substrate, and a top plate, forming a closed system. Both formats typically consist of four essential components: a substrate, an electrode layer, a dielectric layer, and a hydrophobic layer [59]. The substrate is the scaffolding of the chip and mainly plays a supporting role. Commonly used substrate materials include glass, paper, printed circuit boards (PCBs), indium tin oxide (ITO) glass, and other flexible materials [60,61,62]. These substrates are chosen for their durability and compatibility with microfluidic chip fabrication processes. Specifically, glass is reusable, and the droplet movement is relatively smooth. Paper and PCBs, on the other hand, are at the bottom of the cost scale and are easy to process. ITO glass, which is light-transmitting, has high chip precision. The electrode layer conducts electricity, which is used to support the formation of the electric field and the electric drive for droplet manipulation. Heavily doped polysilicon, metals [63], or metal oxides [64] are often used as electrode layer materials. Heavily doped polysilicon is usually prepared by chemical vapor deposition, which is a complex and cumbersome process. Metal materials are usually gold (Au), copper (Cu), aluminum (Al), platinum (Pt), and other chemically stable metals that have the heavy metal feature of strong electrical conductivity. To make the heavy metals and the substrate bond better together, a metal transition layer needs to be added between the heavy metals and the substrate. Metal–oxygen objects, such as ITO, have high conductivity, good mechanical properties, high visible light transmittance, and chemical stability, and are cheap, but the production of electrode patterns is more complex. The dielectric layer is mainly used to accumulate charge so that the droplet can prevent electrode breakdown during the manipulation process. The required voltage for droplet manipulation is closely related to the dielectric constant of the dielectric layer material. SiO_2_ has better insulating properties and more mature processing technology, but its dielectric constant is only 2.7, which requires a large enough voltage to drive the droplet in use. Si_3_N_4_ has a dielectric constant of 7.8, which has high dielectric strength, but there are more particles on the prepared film and the uniformity is not good, which is prone to leakage and makes the dielectric layer break. PDMS is a non-toxic, non-flammable, simple silicone polymer compound with good chemical inertness and insulating properties, and is a widely used material in research. SU-8 has good mechanical properties, insulating properties, optical properties, and chemical properties and is also a material that has been widely used in recent years. Parylene, on the other hand, is often obtained by the chemical vapor deposition method and is chemically stable. Lastly, the hydrophobic layer reduces droplet driving resistance and increases the contact angle of the droplets, enhancing their mobility within the device. Teflon-AF [65] and CYTOP [66] are frequently chosen materials for this layer due to their excellent hydrophobic properties. 

**Figure 1:**
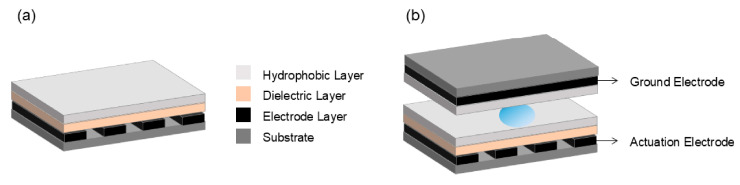
Presentation of the EWOD droplet-driven DMF platform. (**a**) A single-plate DMF device with a hydrophobic layer, a dielectric layer, an electrode layer, and a substrate from top to bottom. (**b**) A two-plate DMF device with detail of the plate acting as the ground electrode and the lower plate as the actuation electrode.

Figure 1 illustrates the EWOD droplet-driven DMF platform. The most basic types of droplet operations achievable by the EWOD platform include transportation, splitting, merging, and dispensing. Studies in the field have shown that droplets on two-plate DMF devices can be dispensed, moved, split, and merged [67,68], whereas droplets on single-plate DMF devices cannot be split. 

### 2.2. Magnetic Force Droplet-Driven DMF

Magnetic digital microfluidics (MDMF) is a specialized branch of DMF technology that utilizes magnetic forces to precisely manipulate droplets [69,70,71,72]. The utilization of a magnetic field can effectively control the magnetic beads suspended in the droplets, which can be used to transport and locate specific biological targets [73]. The magnetic core, a critical component of the magnetic beads, possesses the necessary magnetic properties for the magnetic operation of the beads in different magnetic fields [74,75]. Various pure metals and their oxides are commonly used as core materials. Among these materials, iron oxide (Fe_2_O_3_) is considered to be the most widely used biomaterial due to its excellent magnetic properties, controllable morphology, low cost, and good biocompatibility.

The MDMF consists of an automatic drop control module, a signal-detecting module, and a magnetically controlled digital microfluidic chip. The automatic drop control module is used to manipulate the droplets. The movement of magnetic droplets on surfaces primarily relies on the magnetic force and interfacial resistance [76]. Hence, substrates characterized by low surface tension and minimal adhesion properties are deemed optimal for magnetic droplet manipulation. Polytetrafluoroethylene amorphous fluoropolymer stands out as the standard hydrophobic surface coating material utilized in DMF devices [28]. Signal detection modules are generally associated with a variety of off-the-shelf immunoassay techniques. Digital microfluidic chips consist of a chip plate and a cover plate featuring microwells and microchannels. Microwells serve to contain samples and reagents, while microchannels realize the interconnection of adjacent microwells. MDMF is well established, and the platform is compatible with both permanent magnets, electromagnets, and combined magnetic fields for easy droplet manipulation [77,78,79,80].

Moving permanent magnets is a common method of magnetic field control, and many studies have demonstrated the use of permanent magnets to manipulate droplets in bioanalytical analysis [81,82]. The manipulation of droplets using permanent magnets for magnetic control does not require a power source, gradually becoming a common method for outdoor, power-free immunoassays. However, this method cannot adjust the strength or polarity of the magnetic field. In contrast to permanent magnets, electromagnets can efficiently regulate the strength of the magnetic field by varying the current flowing through them [78]. An electromagnet consists of an iron core and an energized coil [83]. The core is a ferromagnetic material, and the coil is wound around the core. It can concentrate magnetic flux when energized and demagnetize rapidly when de-energized. This suggests that electromagnets offer greater flexibility in adjusting the strength of the magnetic field by modifying the current [84]. Anchored by this analysis, Yang et al. [85] have introduced a droplet manipulation platform that leverages a superhydrophobic electromagnet needle (EMN). This innovative platform utilizes polymethylmethacrylate (PMMA) as the substrate and coats it with a nanosilica-based superhydrophobic agent. By adjusting the power supply, the platform adeptly performs a range of droplet manipulations, including delivery, fusion, magnetic bead extraction, and dispensing. Due to the EMN’s superhydrophobic coating, it can directly link with the magnetic droplets and exert a strong magnetic force on the magnetic beads suspended in them, thus effectively manipulating the droplets without creating wetting or contamination problems. Furthermore, the integration of electromagnets and permanent magnets into a hybrid magnet system creates synergies [86]. On one hand, this hybrid strategy reduces the electromagnetic field current required by a single magnet, thus minimizing the Joule heating effect [87]. On the other hand, it also facilitates the precise tuning of the local magnetic field, providing greater flexibility in the operation of magnetic droplets [88]. The study conducted by Yu et al. [83] integrated permanent magnets and electromagnets to develop an automated robotic system for microfluidic delivery. Their work first drove a permanent magnet with an electromagnetic coil and then drove a biocompatible magnetic droplet through the permanent magnet to quantify the active matrix metallopeptidase (MMP) in human plasma.

**Figure 2:**
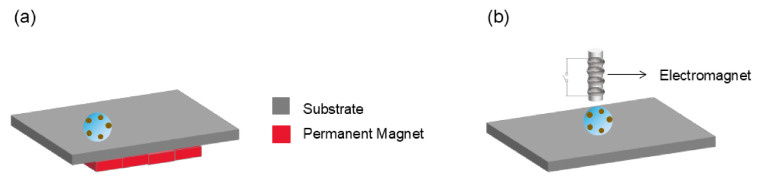
Presentation of MDMF platform. (**a**) Permanent magnet, which is magnetic itself. (**b**) Electromagnet, whose magnetism can be varied. It is magnetic when energized and not when not energized.

Figure 2 presents the MDMF platform, including a permanent magnet and an electromagnet. In the case of the former, the droplet is activated for manipulation when the permanent magnet is near the magnetic droplet and vice versa. In the case of the latter, the switching of the magnetic field is achieved by switching the power supply to the electromagnet, and the magnetic droplet is manipulated in the transformation of the magnetic field.

### 2.3. Surface Acoustic Wave (SAW) Droplet-Driven DMF

SAW control is the emission of surface acoustic waves of different frequencies by the system, which generates an acoustic wave field in the microfluidic chip [20,89]. Acoustic waves possess the capability to generate forces in liquids, thereby enabling the manipulation of suspended particles or liquid media [90,91]. One highly appealing aspect of microfluidic actuation and manipulation via SAW is its remarkable effectiveness in fluid–solid coupling, owing to the concentrated energy near the interface. In 1934, King et al. [92] proposed the concept of particle manipulation in microfluidics using acoustic radiation force, but it did not attract widespread attention. In 1965, White et al. [93] established the basis for the study of SAW devices by transmitting piezoelectric surface waves in the plane of piezoelectric plates. In 1998, Zhu et al. [94] proposed the use of acoustic waves for microfluidic actuation. This research is regarded as the first step in the study of SAW because of the successful excitation of SAW and the driving of microfluidic movement. In 2008, GIRARDO et al. [95] combined acoustic surface waves with microfluidics for droplet generation and control, using SAW to drive droplets to move directionally in microfluidic channels. Subsequently, the rapid development of SAW droplet-driven DMF has enabled the selection and precise manipulation of the driving droplets [96].

**Figure 3:**
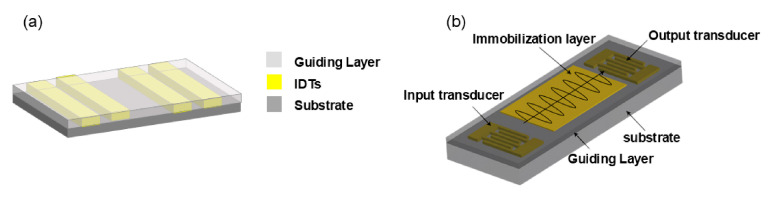
Presentation of the SAW droplet-driven DMF platform, including the guiding layer, IDTs, and piezoelectric substrate, respectively. (**a**) Cross-sectional view. (**b**) Top view. (Adapted with permission from Ref. [98]. Copyright © 2016, Elsevier).

Surface acoustic wave manipulation platforms typically include a piezoelectric substrate and a transducer, the electrode structure of which usually consists of a pair of interdigital transducers (IDTs) in opposite directions that serve as an input transducer and an output transducer [97] (Figure 3).

### 2.4. Other Platforms

**Figure 4:**
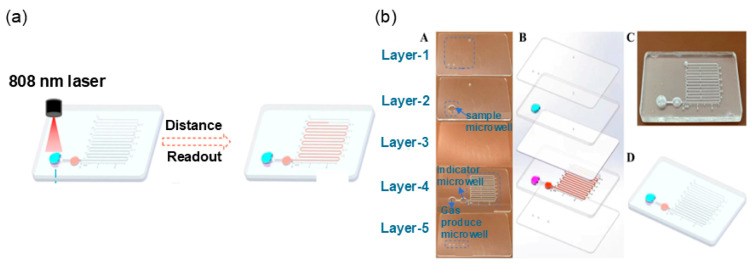
Schematic diagrams of the optical-driven DMF platform. (**a**) Drive schematic of digital microfluidics for optical drives. (**b**) Photographs and 3D schematics of the chips used. (A,B) Exploded view illustration of the μDAD. (C,D) Photograph and 3D schematic of an assembled μDAD. (Adapted with permission from Ref. [99]. Copyright © 2023, ACS).

In addition to the above three classical droplet manipulation platforms, the proposal of new droplet drive methods has led to several new manipulation platforms. The light droplet-driven DMF platform has emerged as a manipulation platform that bears a resemblance to the EWOD platform [99] (Figure 4a,b). It distinguishes itself by replacing the electrode layer with a photoconductive layer that harbors virtual electrodes. Compared to the EWOD platform, it does not require a large number of electrodes and only requires a beam of light for the lossless manipulation of the droplet [100,101]. Additionally, the optically formed reconfigurable virtual electrodes enable parallel manipulation of numerous particles within a specific region of interest across a vast area, surpassing the limitations of conventional electrical methods that rely on fixed electrode patterns [102]. A DMF platform for light manipulation was demonstrated by Loo et al. [103]. Their design incorporates a transparent glass substrate coated with ITO, topped by an amorphous silicon (a-Si) photoconductive layer and an aluminum oxide (Al_2_O_3_) dielectric layer. A notable innovation is the integration of a metal mesh grounding network within the dielectric layer, with droplets situated above the metal mesh. This method effectively replaces the top cover electrode in previous generations of photoelectric wetting devices. This has eliminated the need for the top electrode in conventional photoelectric wetting devices, avoided droplet manipulation on both sides of the platform, and realized direct, visual, and easily adjustable manipulation, thus improving the efficiency of droplet movement and reaction conduct. In addition, surface stresses [104] and the thermo-capillary [105]-based actuation of droplets have also been reported.

## 3. Preparation of DMF

The choice of materials and fabrication techniques largely determines the nature of DMF devices, as they can have a huge impact on fluid flow rate, capillary pressure, wettability, optical properties, biomolecule adhesion, and signal detection [106]. Droplet generation is a prerequisite for subsequent experiments, and different actuation methods have different droplet manipulation principles [107]. In this section, we will summarize the preparation of DMFs in terms of material selection and fabrication techniques and introduce droplet generation and driving using EWOD droplet-driven DMFs as an example.

### 3.1. Materials Selection

The choice of materials not only concerns the quality of the DMF chip but also directly affects the success of subsequent experiments. Therefore, the performance of the material must be considered [108]. First, good durability ensures that the chip can operate stably for a long period under various experimental conditions. Secondly, ease of fabrication facilitates the processing of the structure into specific functions. Thirdly, good transparency guarantees the clarity of signal detection and observation. Fourthly, good biocompatibility is designed to prevent adverse effects on the biological samples. Finally, the potential for surface functionalization provides for diversified functional properties. Currently, commonly used materials include silicon, quartz, glass, polymer, hydrogel, paper, etc.

Silicon is a commonly used material for DMF platform substrate preparation due to its simplicity of fabrication, ease of surface modification, and better mechanical strength and semiconductor properties [109]. Quartz and glass, on the other hand, are widely used in applications requiring high-precision optical detection due to their excellent transparency, low cost, and chemical stability [110]. Polymer materials include thermoplastic polymers, thermoset polymers, and solvent volatile polymers. Typical examples of thermoplastic polymers are polymethylmethacrylate (PMMA) [111]; typical examples of thermoset polymers are polydimethylsiloxane (PDMS) [112]; and typical examples of agent volatile polymers are acrylic acid. They are inexpensive to manufacture, simple to process, and have good transparency. The porous structure of hydrogels neutralizes the water-absorbing and swelling properties, making them an important material. For decades, paper has been increasingly recognized as a promising substrate material for DMF applications because of its light weight, flexibility, low cost, and practicality.

### 3.2. Fabrication Techniques

Different DMF chip fabrication techniques are selected according to the different characteristics of the substrate. The main fabrication methods for silicon materials are photolithography and etching [113]. Photolithography includes multiple steps such as coating, exposure, and development and utilizes photoresists and mask plates to precisely transfer the pattern to the silicon wafer. Etching includes dry etching and wet etching, and typically involves a chemical reaction or physical bombardment to remove portions of the material surface. The common processes for glass and quartz are photoetching and lithography [114]. Photoetching combines the steps of photolithography and etching, where the pattern is first transferred to the material using photolithography, and then the unprotected part of the material is removed by etching. It inherits the high precision of photolithography and the flexibility of etching. The precision and complexity of the latter are much lower than those of photoetching. Polymer DMF chips are mainly fabricated by hot pressing, molding methods, and injection molding methods [115]. Hydrogel DMF chips are mainly prepared by UV laser or 3D printing [116]. The preparation strategy of paper-based DMF chips mainly involves constructing hydrophilic channels on the surface of the paper substrate so that the droplet samples flow directly through the predefined channels. The current processes include inkjet printing, wax printing, screen printing, PDMS printing/soft lithography, photolithography, laser ablation/blasting/engraving, plotter, stamping/pressing, hot embossing, casting, chemical vapor deposition (CVD), CNC (computer numerical control) milling, and flexographic printing [117,118].

### 3.3. Generation of Droplets

Currently, biological contamination remains a difficult problem in in vitro diagnosis. In DMF, these droplets are regarded as independent microreactors, and biological samples are encapsulated within the droplets. This method can effectively protect biological samples from the external environment and avoid cross-contamination between different samples, which is especially important for biological analysis requiring high sensitivity and accuracy [119]. In addition to physical isolation, researchers have found that Pluronic F68 and Pluronic F127 can be added to the droplets to mitigate protein-induced biofouling in immunoassays in DMF devices, as these polymers reduce nonspecific surface adsorption of proteins and other molecules [120,121]. This subsection describes droplet generation and actuation.

In EWOD, the forces affecting droplet motion can be categorized into driving forces and resistance, with the former being the main influence. The Young–Lippman equation is often used to estimate the driving force. When a conductive droplet is located on a substrate, it is in the shape of a spherical cap due to mechanical equilibrium. After a voltage is applied to the substrate, a layer of charge is formed at the interface between the droplet and the insulator, resulting in a decrease in the interfacial tension of the droplet, i.e., a change in the contact angle (*θV*), which leads to the phenomenon of deformation and displacement of the droplet [8]. The Young–Lippman equation describes the relationship between the voltage and the contact angle (*θ*) (eqn) [67,122], where *θ*_0_ and *θ* are the contact angles before and after applying the voltage, *ε*_0_ and *ε_r_* are the free-space permittivity and relative permittivity of the dielectric layer, and *d* is the thickness of the dielectric layer. *γ* denotes the interfacial tension between the droplet and the insulating medium. By switching the voltage between neighboring electrodes “on” and “off”, an interfacial tension gradient and, thus, shear force is generated to drive the droplet.

$$\cos\theta =\frac{\varepsilon_{0}\varepsilon_{r}V^{2}}{2\gamma d}+\cos\theta_{0}$$

**Figure 5:**
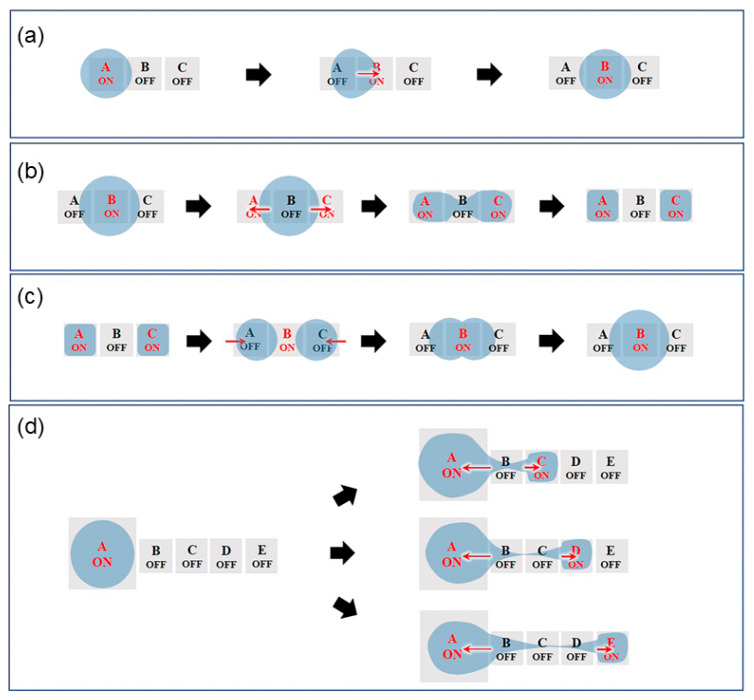
Four types of EWOD-based manipulation of droplets, from top to bottom, are (**a**) transportation, (**b**) splitting, (**c**) merging, and (**d**) dispensing. The gray panels (A–E) represent different voltage units. Red "on" represents voltage on, black "off" represents voltage off. (Adapted with permission from Ref. [123]. Copyright © 2023, RSC).

Currently, the most basic types of droplet operations achievable by the EWOD method include transportation, splitting, merging, and dispensing. Yang et al. summarized four methods of droplet actuation, as shown in Figure 5.

#### 3.3.1. Droplet Transporting

As shown in Figure 5A, when a voltage is applied to electrode unit A, the potentials between electrode units A and B become unequal. When the voltage in electrode unit B increases and the voltage switch in electrode unit A is turned off, the droplet is transported from plate A to plate B.

#### 3.3.2. Droplet Splitting

As shown in Figure 5B, to demonstrate the droplet-splitting process, the droplet is placed on the B electrode unit with voltage applied. When the voltage is removed from the B electrode unit and applied to the electrode units A and C, the contact angle of the droplet becomes smaller, and the driving force causes the droplet to form a “rugby ball” shape on both sides and then droop down in the middle to form a “bottleneck”, which eventually separates into two sub-droplets.

#### 3.3.3. Droplet Merging

As shown in Figure 5C, droplet merging can be achieved by two small droplets transported to the same electrode unit. To demonstrate the droplet-merging process, the droplets are distributed to the A and C electrode units. When the voltage applied to the A and C electrode units is removed and the voltage is applied to the B electrode unit at the same time, the droplets will approach the B electrode unit from both sides and eventually merge into one large droplet.

#### 3.3.4. Droplet Dispensing

Droplet dispensing is the separation of a plurality of small droplets from a large droplet. As shown in Figure 5D, a large droplet is placed in the A electrode unit, and when the voltage of the A electrode unit is turned off and the voltages of the B, C, and D electrode units are turned on sequentially, the droplet separates from the A electrode unit to the B, C, and D electrode units sequentially, and thus a plurality of sub-droplets are generated from the large droplet.

## 4. Digital Microfluidics for Immunoassays

Proteins are crucial biomarkers in the realm of disease detection, monitoring, and treatment. In pursuit of fast and convenient biomarker detection, efforts have been made to construct immunization platforms that enable POCT [124]. Such platforms combine the preparation of the sample and the subsequent examination into one apparatus; they also greatly reduce cross-contamination, simplify complicated experiments, and increase the efficiency of the whole process. Existing immunodiagnostic techniques for POCT rely primarily on the lateral flow immunoassay [125]. This method utilizes capillary forces to drive the flow of the sample across a test strip and to immunoreact with specific antigens or antibodies pre-immobilized on it. The complex bands generated are used for the qualitative assessment of the target substance. It boasts advantages like simplicity, convenience, and swiftness, yet suffers from poor sensitivity, reproducibility issues, and an inability to quantify results. DMF technology, with its broad adaptability, has successfully integrated multiple immunoassay strategies to build powerful analysis platforms. Traditional immunoassays, including sandwich, competitive, direct and indirect methods, have been successfully combined with DMF, demonstrating better sensitivity, specificity and accuracy, and applied in biomarker detection [126]. Of these, the sandwich and indirect methods were more frequently reported. In addition to being used for qualitative analysis, DMF enables precise control of reaction conditions and sample volume, making it well suited for accurate quantitative analysis of target substances. These platforms, especially multi-target immunoassay platforms [127] and automated POCT platforms [28], enable the simplification and optimization of the assay process through the precise control of microfluidic operations. They not only enable the simultaneous detection of multiple targets in multiple samples, significantly improving assay throughput and efficiency, but also dramatically reduce sample and reagent consumption, lowering assay costs. Overall, the emergence of automated POCT platforms has pushed immediate detection to a new level, enabling results to be obtained in a short period of time, meeting the urgent needs of clinical, scientific, and field testing.

Digital microfluidic systems combine sample preparation, reaction, detection, and analysis, so this design philosophy emphasizes advanced integration to ensure optimal performance and efficiency. Consequently, any alterations made to the equipment configuration necessitate a comprehensive review of the original design. This review process, which involves meticulous examination and potential redesign iterations, can be quite costly and time-consuming. Fortunately, in recent years, modular systems have garnered significant attention. Pojchanun Kanitthamniyom et al. [128] proposed a modular digital microfluidic architecture that supports rapid on-demand configuration and reconfiguration. This architecture makes it easier for custom immunoassays, offering a more flexible and efficient approach to microfluidic system design.

This section discusses in detail the recent advances in integrating biomolecular detection technologies on DMF platforms, including colorimetric, direct chemiluminescence, enzymatic chemiluminescence, electrosensory, and other immunoassays. Table 1 summarizes different detection techniques in DMF.

### 4.1. Colorimetric Immunoassay Detection

**Figure 6:**
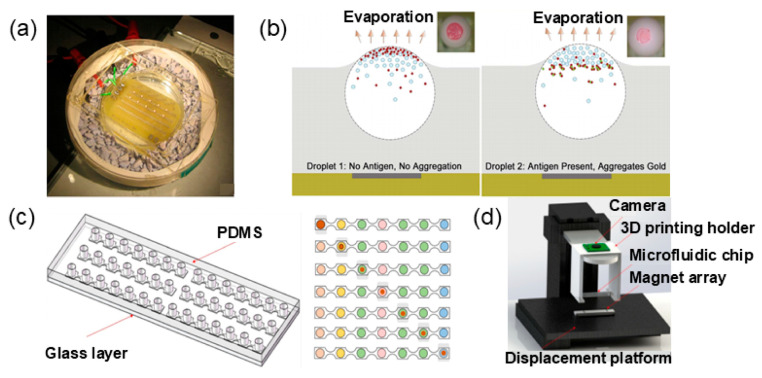
Immunoassay platform based on DMF and colorimetric detection: a, b for qualitative detection; c,d for quantitative detection. (**a**) Experimental setup of the immunoreaction. (**b**) The schematic diagram and microphotograph on the left side show the absence of antigens, while the schematic diagram and microphotograph on the right side demonstrate the aggregation of gold nanoparticles in antigen-containing droplets. (Adapted with permission from Ref. [134]. Copyright © 2007, AIP Publishing). (**c**) The left figure shows the design of the chip. The upper layer is a perforated PDMS channel layer, and the lower layer is a glass support layer; the right figure is a schematic diagram of the CEA detection workflow. From top to bottom are the enrichment of MNPs using magnets; the capture of CEA in the sample by MNPs; PBST washing; the bonding of MNPs and CEA to PS-HRP; the first washing of MNPs by PBST, CEA, and PS-HRP; the second washing of MNPs by PBST, PEA, and PS-HRP; and the HRP-catalyzed color development reaction of the mixed TMB solution, respectively. (**d**) Schematic diagram of the working system, including camera, 3D printing holder, microfluidic chip, magnet array, and display platform. (Adapted with permission from Ref. [48]. Copyright © 2023, RSC).

Colorimetric technology allows qualitative judgment to be made by the direct visual inspection of color changes, which has great potential in POCT applications. Rastogiet et al. [134] employed a method in which gold and latex particles were attached to the antibody, and then ricin was introduced as an antigen. Due to the specific binding of ricin to the antibody, the color change is visible to the naked eye (Figure 6a,b). One of the challenges of qualitative experiments is to ensure the relative independence of the reagents. Once cross-contamination occurs, false positives can easily occur. As a result, researchers have worked to develop platforms that allow various assay reactions to be performed in parallel with each other. The 3D magnetic manipulation strategy [135] is one of the feasible options. This droplet manipulation mode involves contacting and mixing different reagents by wrapping a reagent droplet around a magnetic bead and transporting it vertically through a permanent magnet at the top onto a flat platform, where it then merges with another reagent droplet on the flat surface. This method innovatively addressed the cross-contamination problem of conventional 2D droplet platforms and optimized analytical performance. Their research has demonstrated that two different chemical droplets can produce deposits through chemical reactions, exhibiting potential applications in immunoassays.

However, readings by visual observation are subjective, and there is no standard for quantitative analysis, which lacks credibility. The quantitative analysis of analytes achieved in colorimetric assays is commonly expressed in terms of absorbance. This method is used to measure the absorption intensity of monochromatic light by the change in the absorbance at a particular wavelength and the absorbance at the reference wavelength. Alkaline phosphatase (ALP) and horseradish peroxidase (HRP) are the more commonly used enzymes in colorimetric reactions. For example, Peng et al. [136] carried out a colorimetric enzyme test in a DMF apparatus that produces electricity by converting the mechanical energy pulses of the human finger into a voltage pulse. The enzyme substrates in this case include soluble 5-bromo-4-chloro-3-indolyl phosphate (BCIP) and nitrotetrazolium blue chloride (NBT). Detection of ALP on the antibody results in the formation of purple and blue deposits on the substrate, leading to a marked color change. The active pH range of ALP is 9.5 to 10.5, and HRP shows a higher catalytic efficiency when the reaction system is in an acidic condition. For example, Hu et al. [48] proposed a colorimetric detection method based on an automated immunomagnetic separation platform and a microfluidic chip for the rapid and sensitive detection method of carcinoembryonic antigen (CEA). Immunomagnetic nanoparticles (MNPs) were used to capture CEA in the samples. The CEA detection antibody and HRP were modified on polystyrene (PS) microspheres in the presence of hydrogen peroxide (H_2_O_2_), and 3,3′,5,5′-tetramethylbenzidine (TMB) was used as a signal output for quantification (Figure 6c,d). In addition to these two commonly used enzymes, the proven performance of soybean peroxidase (SBP) [137], a cheaper enzyme that provides a long-lasting chemiluminescent signal, may have the potential to be an ideal alternative to HRP and ALP for immunoassays.

Optical inspection in biomolecular detection typically involves the use of a spectrophotometer, which comprises a light source and an optical detector. Among the commonly employed light source detectors, photodiodes are favored due to their affordability and robustness. In general, the biological analyte is typically placed between the light source and the optical detector. In the case of a transparent DMF device substrate [138], the light can directly pass through and be detected by the optical detector, converting the light signal into measurable output signals. If the substrate is not transparent, however, a hole must be inserted into the substrate to create a light path that connects the light source and the light detector.

Recently, the highly integrated DMF platform and visual colorimetric detection have achieved leapfrog progress in the POCT field, enabling reliable blood specimen testing in remote areas with poor medical conditions. On one hand, the study by Manet et al. [139] allowed for the quantification of target analytes via smartphones. Their research established a novel microfluidic colorimetric immunoassay, utilizing an alternariol monomethyl ether (AME) monoclonal antibody (GNP-MAB) modified with gold nanoparticles (GNPs) as a detection probe for the identification of varying concentrations of AMEs. Smartphone imaging was employed to monitor the color change of the GNPs, allowing for the measurement of AME concentrations as low as 200 pg/mL within a 15 min timeframe. On the other hand, Jackson et al. [140] have developed an alternative hydrophobic membrane that can be used to bind antibodies. When placed over the OpenDrop array of the testing platform, it can be utilized for ELISA testing. Laboratories equipped with microfluidic platform conditions solely require the purchase of compatible kits for the quantitation of diverse analytes. Furthermore, digital immunoassay systems based on artificial intelligence (AI) hold considerable potential for the screening and analysis of various clinical diseases. For example, Zhao et al. [50] introduced a multidimensional digital immunoassay technique that involves the encoding of microparticles and AI-based decoding. This microfluidic digital immunoassay technique is capable of the simultaneous detection of several inflammatory markers and antibiotics in 30 min, showing high sensitivity and a broad detection range, which makes it possible to analyze the analyte from pg/mL to g/mL. As a cutting-edge multiplexed biosensing technology, it has broad application prospects in the field of intelligent bioimmunoassays.

### 4.2. Chemiluminescence Immunoassay (CLIA) Detection

**Figure 7:**
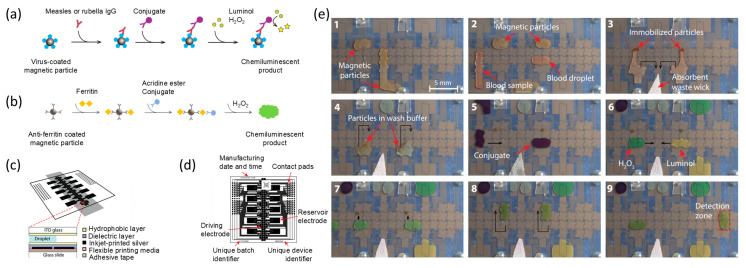
Cartoon schematic of the chemiluminescent reaction of the chemiluminescent agents lumino and acridinium ester. (**a**) Paramagnetic particles coated with measles or rubella virus antigens were incubated with the sample. Anti-measles or anti-rubella IgG bound to the pellet. The particles were washed and then incubated with anti-human IgG-HRP coupling. The particles were washed again and then exposed to a mixture of lumino and H_2_O_2_. Enzymatic turnover of the product generated a chemiluminescent product. (Adapted with permission from Ref. [141]. Copyright © 2018, AAAS). (**b**) Magnetic particles coated with anti-ferritin and ferritin were incubated for 15 min. These particles were washed twice and then exposed to anti-ferritin acridine ester coupling. H_2_O_2_ was introduced into the reaction reservoir and generated the chemiluminescent signal. (**c**) Design and assembly of an integrated microfluidic chip for chemiluminescence immunoassay. (Adapted with permission from Ref. [141]. Copyright © 2018, AAAS). (**d**) Design of an integrated DMF device for chemiluminescence immunoassay. (Adapted with permission from Ref. [141]. Copyright © 2018, AAAS). (**e**) Realistic response to the manipulation process on the instrument. Black arrows indicate the direction of droplet movement. Red arrows indicate different reagents and manipulations. 1 Add particle suspension; 2 Added blood sample and fuse with particles; 3 Immobilized particles; 4 Washed; 5 Added antibody conjugate and incubate with particles; 6 Added luminescent material dropwise and mixed; 7 Separate; 8 Incubated particles with luminol and H_2_O_2_; 9 Moved particles to detection zone. (Adapted with permission from Ref. [141]. Copyright © 2018, AAAS).

Chemiluminescent agents play a direct role in the luminescence reaction during the luminescence immunoassay process. These agents possess a unique chemical structure that produces luminescence, enabling them to label antigens or antibodies directly. Direct chemiluminescence offers rapid results and good reagent stability, but it exhibits slightly lower sensitivity than enzymatic luminescence. Luminol and its derivatives were first used in CLIA. Luminol can be oxidized by oxidizing agents under alkaline conditions, radiating light at a wavelength of 425 nm. Alphonsus H. C. Ng et al. [141] employed microfluidics to detect measles and rubella immunoglobulin in remote environments. In this work, the target measles/rubella immunoglobulin was captured with magnetic beads coated with a capture antibody. Then, anti-measles/anti-rubella antibodies and anti-human immunoglobulin coupled with HRP were introduced. HRP causes oxidative degradation of luminol and emits light at a wavelength of 428 nm, thus allowing chemiluminescent detection for quantification (Figure 7a). However, the luminescence of the luminol system is essentially of a flashing type, and the signal is not that strong. When luminol is coupled with antibodies to form a detection antibody conjugate, the luminescent efficiency decreases. In order to improve the luminescence efficiency of the luminol system, Zeng et al. [142] proposed a coupled grounding model and constructed a unipolar voltage-driven EWOD device. This design not only simplified the structure of the chip and control circuitry but also allowed for the fluorescence focusing of spherical droplets, thereby increasing the detected light signal and improving detection sensitivity. In contrast, acridinium ester is a highly luminescent chemical agent that exhibits high activity when labeled with antibodies. The automated microfluidic chemiluminescence immunoassay platform reported by Min et al. [129] can detect the photon signal of acridinium ester under alkaline conditions at a wavelength of 430 nm, thus enabling the quantification of biological analytes. Acridine esters have high luminescence efficiency when oxidized by H_2_O_2_ under alkaline conditions. The excited state product N-methylacridone (NMP) serves as the luminescent body in this luminescence reaction system (Figure 7b). These chemiluminescent substances belong to the flash type and reach their maximum luminescence intensity around 0.4 s after the addition of a luminescence-inducing reagent.

Due to its great sensitivity and selectivity in a range of assays, the fluorescence approach employed in chemiluminescent assays (including CLIA with CLEIA below) is widely used. The integration of chemiluminescence detection into DMF platforms usually requires photomultiplier tubes (PMTs) to amplify the chemiluminescence signal [45,129,143]. This is because small volumes of samples and reagents will have low signals. Additionally, PMTs can effectively convert photons into electrons and then amplify these electrons multiple times, thereby enhancing the signal, minimizing background noise, and facilitating sensitive detection with superior signal-to-noise ratios. Chemiluminescent detection as a viable technique for POCT applications benefits from the ability of detection devices (commercial PMTs) to be simply interfaced with DMF platforms, but this method requires a light source to provide the excitation wavelength and detection of the emission wavelength, and spectral overlap becomes a significant challenge for multiplexed protein detection. The selection of specific chemiluminescent probes to produce enzyme–substrate luminescent reactions at specific wavelengths [144] and physical spatial separation of signals on sensors [145] can solve the problem of spectral overlap in multiple biomarker detection.

### 4.3. Chemiluminescence Enzyme Immunoassay (CLEIA) Detection

Chemiluminescent enzyme immunoassays work by using an enzyme that has been coupled to an antibody to catalyze the emission of light from a substrate. The enzyme is the key to the technique, and the catalysis of the enzyme requires a certain reaction time. Additionally, the introduction of external variables may have an effect on the activity of catalase. However, the technique is highly sensitive and well suited for automation, which facilitates high-throughput screening and consistency of results. Currently, this methodology has been widely used in testing and is utilized for a wide range of biomarker assays. Sista et al. [79] combined digital microfluidics with ELISA for the detection of interleukin-6 (IL-6) and insulin in 7 min. In this approach, two droplets were created: one containing the target analyte and the other containing a capture antibody conjugated to a magnetic bead and encapsulating a detection antibody coupled to ALP. When these two droplets merged, a sandwich structure was formed, and the ALP on the detection antibody reacted with the alkaline phosphatase substrate-5 (APS-5) to generate a chemiluminescent signal. The following year, the authors utilized a microfluidic platform reliant on magnetic manipulation to perform chemiluminescent immunoassays and developed a small instrument for POCT. Magnetic beads in the droplets bind to the capture antibody. By moving the permanent magnet, the beads can be aggregated, released, transferred, and mixed between the droplets, resulting in the capture and detection of antibodies binding to the target protein. Notably, this platform enables rapid immunoassay of cardiac troponin I (cTnI) in less than 8 min [146]. Later, Lu et al. [78] proposed an integrated platform for chemiluminescence immunoassay based on adjustable electromagnets with magnetic drive. Permanent magnets were used to increase the magnetic flux density by orders of magnitude, and adjustable electromagnets could be used for the extraction of magnetic beads and the control of droplet motion. This combination of permanent magnets and electromagnets, as mentioned earlier, reduced the Joule heat generated during maneuvering and also allowed for more flexible droplet manipulation. The platform automated the entire analysis in 40 min and could detect H_1_N_1_ at 0.032 IU/mL.

**Figure 8:**
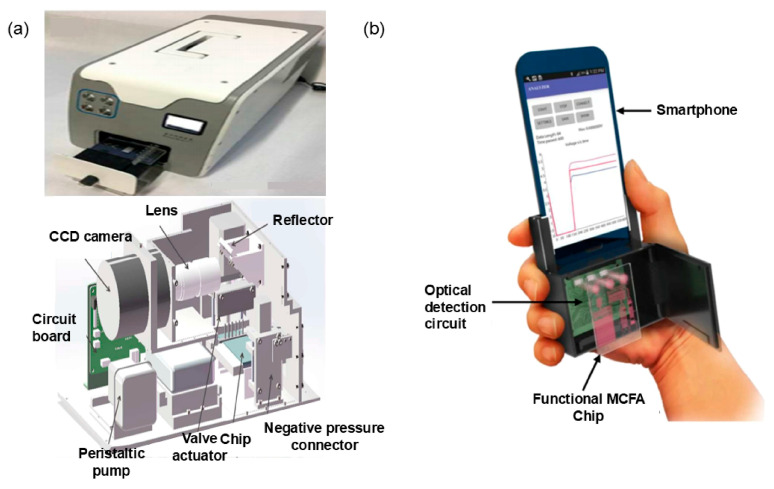
Immunoassay platform based on DMF and CLEIA detection. (**a**) Photograph of a miniaturized, automated chemiluminescent immunoassay analyzer. The main components of the instrument are a CCD camera, lens, reflector, circuit board, peristaltic pump, valve actuator, and negative pressure connector. (Adapted with permission from Ref. [130]. Copyright © 2017, RSC). (**b**) Schematic diagram of a chemiluminescent immunoassay analyzer developed based on a smartphone (adapted with permission from Ref. [148]. Copyright © 2020, Springer Nature).

To meet the objectives of POCT, the technology for incorporating enzyme chemiluminescent assays onto DMF platforms has been developed, honed, and enhanced. On one hand, there is a proliferation of small, portable microfluidic chemiluminescence immunoassay analyzers. In this portable digital microfluidic platform [130], known as an LOC, individual reservoirs of the chip are preloaded with different reagents. In the closed state, mechanical valves on the chip allow the reagents to be isolated from the microchannels. After adding clinical samples, the chip is placed into the detection instrument, and the valves on the chip are opened according to a preset program in the instrument. This allows the samples to be released from the microchannels, and the volume of reagents used for the chemiluminescent immunoassay can be controlled by adjusting the size and opening time of the valve apertures. This type of digital microfluidic platform is very simple to operate. After inserting the chip into an adapter and loading it onto the platform, the user is prompted to choose from a list of pre-stored procedures via the touch screen and initiate the test by pressing the GO button (Figure 8a). Quantification of the target analyte can be achieved with this simple operation. On the other hand, using smartphones to access test results is a hugely promising approach for high-volume disease screening in remote areas. As mentioned before, smartphones can be used as signal readers. The luminescent signal from the chemiluminescent system can be captured by a mobile phone’s camera and analyzed for sample data using customized software (Samsung Galaxy SIII, Android Version 4.2.2) (Figure 8b). This achieves the goal of detection without the need for specialized optical instruments [147].

### 4.4. Electrochemical Detection

Electrical biosensors are highly resilient, easily miniaturized, and perfect for POCT. In electrical biosensors, transducers are essential components that translate biometric events into quantifiable output signals like conductance and current [149]. Electrical biosensors are generally divided into three categories: electrochemical biosensors, field effect transistor (FET) biosensors, and impedance biosensors, depending on how the electrical signal is measured.

**Figure 9:**
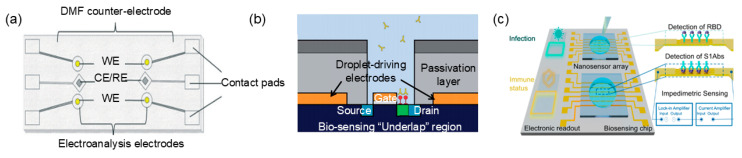
Schematic of the analysis of biomarkers using electrochemical detection, including electrochemical biosensors, field effect transistor biosensors, and impedance biosensors. (**a**) Electrochemical biosensor with working electrode (WE) and counter/reference electrode (CE/RE). (Adapted with permission from Ref. [47]. Copyright © 2014, RSC). (**b**) Field effect transistor biosensor with source, drain, and gate. (Adapted with permission from Ref. [132]. Copyright © 2012, RSC). (**c**) An impedance nanobiosensor based on crossed-finger gold nanowires. (Adapted with permission from Ref. [153]. Copyright © 2023, ACS).

A subtype of electrical biosensors called electrochemical biosensors uses quantitative data to quantify the rates of electron transfer linked to oxidation or reduction reactions at the electrode surface. Each of these biosensors, which are built into the top plate of the apparatus, usually has a working electrode (WE), a counter electrode (CE), and a reference electrode (RE) (Figure 9a) [47]. These electrodes can be precisely deposited on the glass substrate of DMF devices using photolithography and ion beam deposition techniques. However, the fabrication procedures for these methods are cumbersome, which limits the mass production of the devices.

In electrochemical sensors, the number of probes that can be accommodated by the sensing electrodes is the main determinant of the detection limit of the sensor. Therefore, increasing the surface area of the electrodes can achieve the goal of having more molecular probe-binding sites. The team of Wheeler [150] integrated nanostructured microelectrodes (NMEs) into a DMF platform, which has a corrugated surface that facilitates an increase in the number of probe-binding sites on the electrodes. These electrodes exhibited a higher peak signal magnitude during oxidation compared to planar electrodes. Rubella virus (RV) was detected at 0.07 IU/mL using this platform. In this device, both the top plate and the bottom plate consisted of glass. The bottom plate included a Cr DMF driver electrode covered by a layer of polystyrene-C and hydrophobic Fluoro-Opal insulation. The top plate consisted of three conductive layers (ITO, Cr, and Au), an SU-8 insulating layer, and a FluoroPel diagrammatic layer. However, the instability and low current response of ITO electrodes on glass hinder their use in immunoassays. The research by Nsabimana et al. [131] effectively addresses this issue. They utilized ITO electrodes on polyethylene terephthalate (PET) film as the top plate of the DMF. This approach offered higher carrier concentration, faster mobility, and lower resistivity for ITO, resulting in superior conductivity. On this platform, the H_5_N_1_ antigen in human serum was detected by the magnetic bead immunoassay technique. This method exhibited a remarkably low detection limit, reaching as low as 18 ng/mL.

FET biosensors, a subclass of electrical biosensors, quantify interactions by measuring current or potential across a semiconductor. FETs typically consist of three elements: the source, the drain, and the gate. Their operational sequence initiated with a charge that originated at the source, proceeded through the gate, and concluded at the drain (Figure 9b). The gate of Bio FETs was equipped with a biometric element that was fixed for molecular recognition. When charged biomolecules bound to the gate, the distribution of charge changed, resulting in a change in conductance on the gate that can be processed and examined. Choi et al. [132] integrated an FET biosensor into a single-plate DMF device on a silicon substrate. When a droplet containing an avian influenza virus moved onto the FET sensing electrode, it was detected as an avian influenza virus droplet. Then, as the droplet containing the avian influenza virus moved on to the FET sensing electrode and bound to the silicon-binding protein (SBP-Ala antigen) on the surface of the “underlap region,” the drain current decreased, indicating the presence of avian influenza. This assay was completed in seconds and demonstrated a detection limit of 3.67 pg/mL.

Impedance biosensors measure the electrical impedance of an interface. When the target analyte binds to the sensor surface, it produces an impedance effect on the alternating current (AC), allowing for the quantification of the analyte through impedance changes (Figure 9c). Kechadi et al. [151] introduced a novel two-electrode contactless structure integrated into a DMF platform for protein quantification. This structure was composed of impedance-measuring electrodes on a borosilicate glass substrate, with the sensing electrode located between two neighboring drive electrodes. In their study, poly-para-xylene C and polytetrafluoroethylene AF1601 were used to provide electrode arrays with electrically insulating and hydrophobic surfaces. However, the hydrophobic nature of the electrode arrays posed challenges in manipulating droplets on the hydrophilic biosensor surface. To address this issue, Mashraei et al. [152] introduced a novel two-ended fractal electrode serving as both the driving and sensing electrode, enhancing droplet transport and facilitating manipulation on the biosensor surface. They immobilized anti-CRP antibodies on fractal electrode devices for the quantification of CRP in human serum.

### 4.5. Other Immunoassays

**Figure 10:**
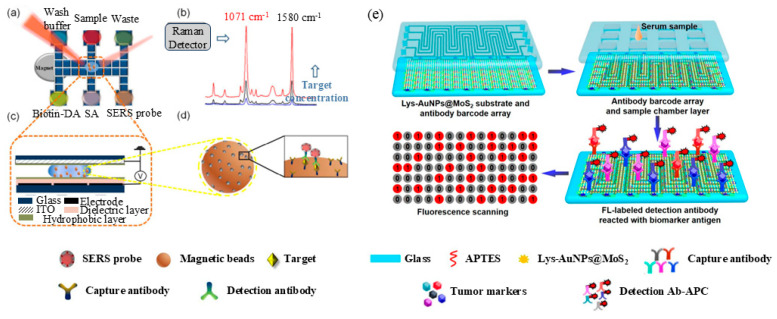
Schematic representation of SERS and SPR immunoassays. (**a**) Illustration of DMF SERS method. (**b**) Two characteristic Raman peaks of 4-MBA. (**c**) Side view of DMF chip. (**d**) Immunocomplex functionalized with SERS tags on magnetic beads. (Adapted with permission from Ref. [44]. Copyright © 2018, ACS). (**e**) Lys-AuNPs@MoS2 substrate, where the MoS2 nanocomposite produces localized surface plasmon resonance (LSPR). (Adapted with permission from Ref. [133]. Copyright © 2022, ACS).

In addition to the above detection methods, surface-enhanced Raman scattering (SERS) is also a popular integration technique [154]. SERS stands out among other detection techniques for its exceptional sensitivity. This arises from its dual capability of amplifying electric fields through plasmonic effects and chemically enhancing the Raman scattering of analytes, even at trace concentrations. Essentially, SERS is the phenomenon of enhanced Raman scattering observed when a laser beam is applied to a molecule adsorbed on a metallic nanostructure. Generally, the enhancement is caused by the coupling between the incident light and the localized surface plasmon resonance (LSPR) on these nanostructures [155]. Wang et al. [44] presented a SERS-based DMF immunoassay for the determination of H_5_N_1_ avian influenza virus in human serum. In this approach, a core–shell nanostructure served as SERS labels, with 4-mercaptobenzoic acid (4-MBA) embedded as a Raman reporter molecule. These SERS tags were conjugated to detect antibodies, allowing for the specific labeling of the captured H_5_N_1_ virus on magnetic beads. Subsequently, the SERS spectrum underwent analysis, with the scattered light being channeled into a charge-coupled device (CCD) detector. This allowed for the precise quantification of virus concentration through Raman scattering. The resulting platform demonstrated remarkable efficiency and sensitivity in detecting the H_5_N_1_ avian influenza virus in clinical samples. It achieved a detection limit of 74 pg/mL while utilizing a mere 30 mL of sample (Figure 10a–d).

Surface plasmon resonance (SPR) is another technology that can be seamlessly integrated into DMF devices. This integration takes advantage of the traditional optical phenomenon where SPR and plasma resonate with each other when evanescent waves are generated in different media. It enables the development of biosensing analysis techniques for the investigation of biomolecular interactions and the real-time detection of immune complex formation. SPR offers significant advantages, as it does not necessitate the addition of fluorescent substrates, enzymes, luminophores, or other labeling agents (Figure 10e). Chuang et al. [156] reported a membrane-based microfluidic device integrated with a surface plasmon resonance (SPR) sensor for easy-to-use and cost-effective multistep quantitative analysis. The innovation of this device lies in the design of separating the absorbing pad from the transferring membrane, thus effectively solving the past problem of liquid propagation not being able to be terminated due to the absorbing pad not being sufficiently wetted by the liquid, and thus realizing the precise control of the liquid flow.

## 5. Challenges and Limitations

DMF has great potential to be applied in immunoassays [157]. However, there are some challenges and limitations of DMF in practical applications. Firstly, achieving in-chip high-throughput detection requires high-chip precision, complicating the manufacturing process and potentially increasing costs. This places greater demands on the choice of materials, fabrication techniques, and programming of the system. In the testing process, the materials selected must be inert and must not interfere with the experimental results, and the fabrication techniques used must have precise parameters to ensure the realization of the complex structure of the chip [158]. In addition, the realization of high-throughput detection requires complex programming of the drive system [159]. Secondly, current optical technologies are predominantly relied upon for detection methods, yet they often demand bulky and expensive optical instruments. This creates a challenge for laboratories that operate with limited resources. Therefore, exploring portable signal readout devices integrated with DMFs is warranted. For instance, utilizing imaging software [160] on smartphones facilitates detection in areas with limited resources. Moreover, it is difficult to integrate inspection instruments with DMF equipment, which requires size compatibility, process compatibility, and so on [161]. Lastly, the automation of sample injection and removal within the chip remains inadequate. While disposable chips could offer a solution, they might increase costs. Conversely, chip reuse could lead to biofouling, potentially resulting in false positives due to sample contamination.

**Figure 11:**
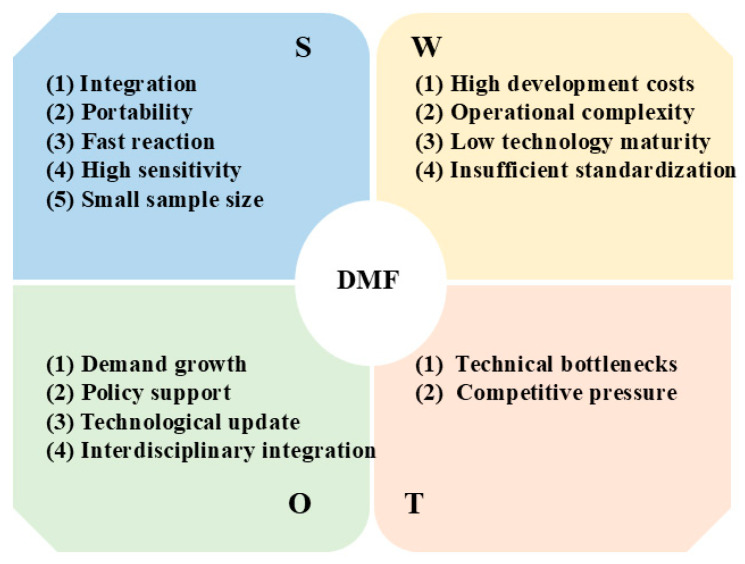
SWOT analysis for DMF.

In order to provide a more in-depth basic understanding of DMF, a SWOT analysis has been conducted here, as shown in Figure 11.

## 6. Conclusions and Outlook

To conclude, various DMF platforms and their applications to immunological analysis are discussed in this review. With the advent of technologies such as 3D printing and rapid prototyping, DMFs are becoming cost-effective and more accessible in bioengineering and medical application environments. In addition to the EWOD, magnetic, and SAW-based manipulation platforms presented in this paper, other emerging drive platforms are currently under refinement. Based on the application of DMF in immunoassay, the detection of protein biomarkers can reach the level of single-molecule detection and achieve a detection limit as low as the order of attomolar, which can detect proteins that cannot be detected by traditional clinical laboratories. This technology has a significant impact on predictive disease assessment and prevention.

At present, the combination of optics and electronics with DMF technology has made it possible to detect biomarkers with unprecedented sensitivity, which has great potential for future POCT. However, the analysis of complex samples within these systems encounters challenges in terms of stability and specificity. Mass spectrometry, on the other hand, offers invaluable insights into the chemical composition of target samples. Therefore, the integration of DMF equipment with mass spectrometry analyzers, aimed at minimizing sample loss and contamination, has emerged as a preeminent trend in the field. The anticipated integration of this technology is poised to profoundly transform the realm of biomarker detection, resulting in the attainment of more precise and dependable diagnostic results.

**Figure 12:**
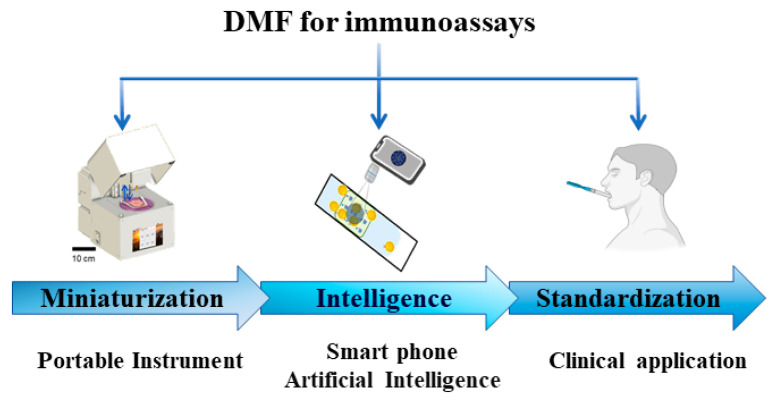
Future prospects of DMF.

Regarding the application of DMF in immunoassays, there is considerable untapped potential beyond its integration with ELISA. It includes the construction of human organoids and the investigation of immune factor interactions within the human immune microenvironment. Furthermore, the amalgamation of DMF, ELISA, and computational technologies represents a promising research avenue toward smarter and more efficient detection. Figure 12 shows the future prospects of DMFs, moving towards miniaturization, intelligence, and standardization. Overall, future endeavors in this domain are anticipated to continually advance DMF’s role in immunoassays, contributing significantly to progress in biological, medical, and clinical applications.

## Figures and Tables

**Table 1:** Summary and comparison of different detection methods.

Detection Methods	Merits	Drawbacks	Applications	Limit of Detection	Ref.
Colorimetry	Easy to use Inexpensive Highly intuitive	It cannot be used for colorless compounds	Detection of inflammatory markers	CRP: 2.39 pg/mL PCT: 2.12 pg/mL IL-6: 0.48 pg/mL	[50]
Direct chemiluminescence	Fast response Easy to use	Difficult to control luminous intensity accurately	Detection of protein marker	Ferritin: 2.55 ng/mL	[129]
Enzymatic chemiluminescence	High sensitivity Wide detection range Proven technology	Enzymes require high reaction conditions Difficult to undergo multiplex detection	Detection of infectious virus	H_1_N_1_: 0.032 IU/mL	[78]
			Detection of hormones	Testosterone: 0.45 ng/mL	[130]
Electrosensory	High sensitivity and specificity Multi-parameter detection capability Real-time analysis	Requires specific working electrode Detectors are disposable	Detection of infectious virus	H_5_N_1_: 0.6 ng/mL avian influenza virus:3.67 pg/mL	[131,132]
Surface-enhanced Raman scattering (SERS)	High sensitivity Non-destructive Simultaneous detection of biomolecules	Low signal-to-noise ratio Long signal collection times	Detection of infectious virus	H_5_N_1_: 74 pg/mL	[44]
Surface plasmon resonance (SPR)	No label required Real-time monitoring Fast response	High technical threshold High equipment cost	Detection of inflammatory factors and cardiovascular biomarkers	IL-6: 0.24 pg/mL PCT: 10.3 pg/mL NT-BNP: 9.57 pg/mL CRP: 6.18 pg/mL CTNI: 19.37 pg/mL CTNT: 38.29 pg/mL	[133]

## Abbreviations

Full Name	Abbreviation
Alkaline phosphatase	ALP
Alkaline phosphatase substrate-5	APS-5
Alpha-fetoprotein	AFP
Alternariol monomethyl ether	AME
Alternating current	AC
Alumina	Al_2_O_3_
Artificial intelligence	AI
Carcinoembryonic antigen	CEA
Cardiac troponin I	cTnI
Chemical vapor deposition	CVD
Chemiluminescence enzyme immunoassay	CLEIA
Chemiluminescence immunoassay	CLIA
Chemiluminescence immunoassay	CLIA
Computer numerical control	CNC
Counter electrode	CE
Digital microfluidics	DMF
Electromagnet needle	EMN
Electrowetting on dielectric	EWOD
Enzyme-linked immunosorbent assay	ELISA
Field effect transistor	FET
Fluorescence immunoassay	FIA
Granulocyte-macrophage colony-stimulating factor	GM-CSF
Horseradish peroxidase	HRP
Hydrogen peroxide	H_2_O_2_
In vitro diagnostics	IVD
Indium tin oxide	ITO
Interdigital transducers	IDTs
Interleukin-6	IL-6
Iron oxide	Fe_2_O_3_
Lab-on-a-chip	LOC
Limit of detection	LOD
Magnetic digital microfluidics	MDMF
Micro Total Analysis System	µTAS
Nanostructured microelectrodes	NMEs
Nitro blue tetrazolium chloride	NBT
N-methylacridone	NMP
Point-of-care testing	POCT
Polydimethylsiloxane	PDMS
Polyethylene terephthalate	PET
Polymethylmethacrylate	PMMA
Polystyrene	PS
Printed circuit boards	PCBs
Prostate-specific antigen	PSA
Radioimmunoassay	RIA
Reference electrode	RE
Rubella virus	RV
Silicon dioxide	SiO_2_
Silicon nitride	Si_3_N_4_
Soybean peroxidase	SBP
Surface acoustic wave	SAW
Surface plasmon resonance	SPR
Surface-enhanced Raman scattering	SERS
Three-dimensional	3D
Tumor necrosis factor-α	TNF-α
Working electrode	WE
γ-Interferon	INF-γ
3,3′,5,5′-Tetramethylbenzidine	TMB
5-Bromo-4-chloro-3-indolyl phosphate	BCIP
